# “When emotions teach”: a multilevel moderated mediation model of teacher emotion display, classroom climate, and student emotional intelligence on academic risk-taking

**DOI:** 10.3389/fpsyg.2026.1882004

**Published:** 2026-09-17

**Authors:** Yuyang Shen, Hongmei Hou, Haibo Zhang

**Affiliations:** 1 University Office, Sichuan Technology and Business University, Meishan, Sichuan, China; 2 School of Digital Economy and Management, Sichuan Technology and Business University, Meishan, Sichuan, China

**Keywords:** academic risk-taking behavior, classroom climate, multilevel modeling, student emotional intelligence, teacher emotional display

## Abstract

**Introduction:**

This study examines the association between teacher emotional display and students’ academic risk-taking behavior, focusing on the mediating role of classroom climate and the moderating role of student emotional intelligence.

**Methods:**

Data were collected from 419 undergraduate students nested within 68 classrooms at Chinese universities using a three-wave, time-lagged design. Multilevel structural equation modeling was employed to test the hypothesized relationships.

**Results:**

Teacher emotional display was positively associated with academic risk-taking behavior both directly and indirectly through classroom climate. The relationship between classroom climate and academic risk-taking was stronger among students with higher emotional intelligence. Moreover, the indirect effect of teacher emotional display on academic risk-taking through classroom climate was more pronounced at higher levels of emotional intelligence, supporting a moderated mediation model.

**Discussion:**

The findings integrate emotional, environmental, and individual factors into a multilevel framework of academic risk-taking. They highlight the importance of emotionally expressive teaching, supportive classroom climates, and students’ emotional intelligence in fostering adaptive learning behaviors.

## 1 Introduction

Learning environments are not solely defined by instructional content; they are also characterized by the emotional signals exchanged between teachers and students. Classrooms function as dynamic social systems in which emotions are associated with attention, motivation, and behavioral engagement. Prior research has increasingly emphasized that students’ willingness to participate, persist, and take initiative is deeply embedded in the emotional tone of the classroom (Gezer, 2023; Olivier et al., 2024). Among these behaviors, academic risk-taking—defined as students’ readiness to engage in challenging tasks despite the possibility of failure (Clifford, 1991)—has gained attention as an important correlate of deep learning and intellectual growth. Students who are willing to take risks are more likely to explore complex problems, ask questions, and develop higher-order thinking skills. However, such behavior is inherently uncertain and often requires a supportive environment that reduces fear of negative evaluation.

Within this context, teacher emotional display emerges as a central yet underexplored factor associated with student behavior. According to Ma et al. (2023), teachers’ expressions of enthusiasm, care, frustration, or disappointment act as salient cues that signal expectations and define the emotional climate of the classroom. Existing studies suggest that positive emotional displays by teachers are associated with greater student engagement and motivation, whereas negative or inconsistent expressions may inhibit participation (Brown et al., 2023; Cheng et al., 2022; Wang et al., 2023; Zhang and Jiang, 2023). Nevertheless, much of the literature has focused on direct relationships between teacher emotions and student outcomes, often overlooking the mechanisms through which these relationships unfold. In particular, there is limited understanding of how teacher emotional display is associated with the development of collective classroom experiences and, subsequently, students’ behavioral responses.

In this milieu, classroom climate provides a compelling explanatory pathway. It reflects students’ shared perceptions of support, cooperation, and psychological safety within the classroom (Wang et al., 2025). Further, a positive climate fosters trust and reduces anxiety, enabling students to experiment, make mistakes, and engage more actively in learning processes. Prior research has demonstrated that classroom climate is associated with various academic outcomes, including motivation and achievement (Doll and Dooley, 2024; Peiris et al., 2022; Wang and Wang, 2024). However, its role as a mediating mechanism linking teacher emotional behavior to academic risk-taking remains insufficiently examined. Addressing this gap is important for advancing a more nuanced understanding of how emotional and environmental factors are jointly associated with student behavior.

At the same time, students are not passive recipients of classroom experiences. Individual differences play a crucial role in determining how students interpret and respond to emotional cues. Student emotional intelligence, defined as the ability to perceive, understand, and regulate emotions (Kawashima et al., 2025; Wong and Law, 2002), represents a key personal resource in this process. Students with higher emotional intelligence are better equipped to manage anxiety, interpret social signals, and adapt their behavior in emotionally charged situations. Although previous studies have established the benefits of emotional intelligence for academic performance and well-being (Jahan et al., 2022; Ogbeta, 2022; Zhou et al., 2024), its role as a boundary condition influencing the effectiveness of classroom climate has received limited attention. Understanding this moderating role is essential for capturing the interplay between environmental and individual factors.

In the present study, emotional contagion theory, control-value theory, and social cognitive theory jointly provide the theoretical foundation for the proposed framework. Emotional contagion theory explains how teachers’ recurrent emotional displays may spread across students and become embedded in a shared classroom climate (Herrando and Constantinides, 2021). On the other hand, control-value theory further suggests that a supportive climate may strengthen students’ perceived control and reduce the anticipated emotional cost of failure, thereby encouraging academic risk-taking (Pekrun, 2006). However, students may differ in their ability to interpret and utilize these contextual signals. Consistent with social cognitive theory, emotional intelligence represents a personal capability that may determine how effectively students translate a supportive classroom environment into adaptive behavior (Bandura, 1986). Together, these perspectives explain how teacher emotional display is associated with academic risk-taking through classroom climate and why this indirect relationship may vary according to students’ emotional intelligence.

Building on these perspectives, the present study develops a multilevel framework that integrates teacher emotional display, classroom climate, and student emotional intelligence to explain academic risk-taking behavior (Figure 1). By conceptualizing teacher emotional display and classroom climate at the classroom level and student emotional intelligence and academic risk-taking at the individual level, the study captures the hierarchical nature of educational settings. Its theoretical contribution lies in specifying a conditional multilevel process in which teachers’ emotional displays are associated with students’ academic risk-taking through a shared classroom climate, while the strength of this cross-level relationship varies according to students’ emotional intelligence. This approach moves beyond isolated direct relationships by explaining both how and when classroom emotional processes are associated with adaptive student behavior.

**Figure 1:**
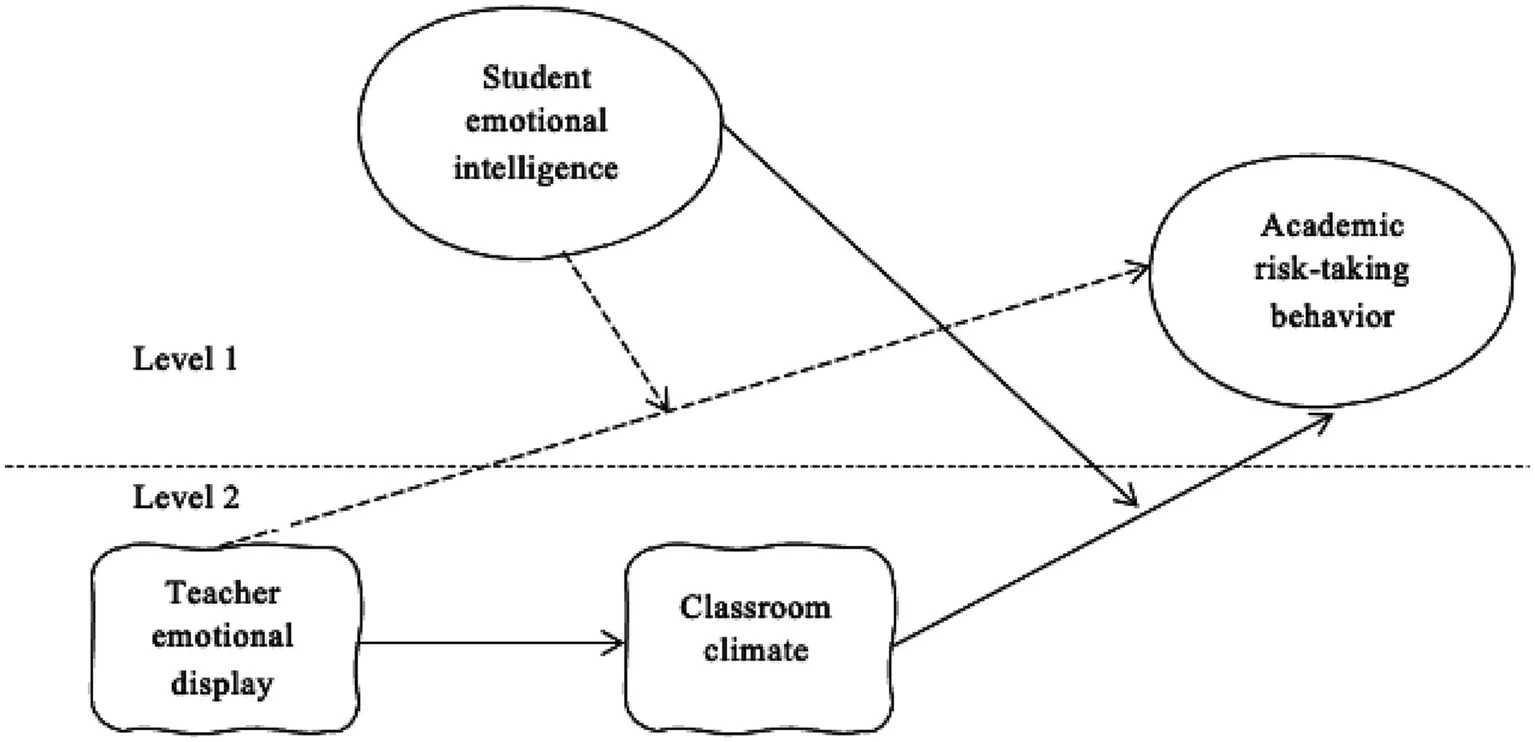
Conceptual model. Rectangular boxes represent Level 2 classroom-level constructs, whereas oval shapes represent Level 1 student-level constructs. Arrows indicate the hypothesized relationships, and the arrow directed toward another relationship represents the proposed moderation effect.

## 2 Literature review

### 2.1 Theoretical framework

The proposed framework draws on emotional contagion theory, control-value theory, and social cognitive theory, which provide complementary explanations for affective transmission, achievement-related behavior, and person–environment interaction. Emotional contagion theory proposes that observing another person’s verbal and non-verbal emotional expressions may produce affective convergence within dyads and groups. In educational settings, research has connected teachers’ expressed emotions with students’ emotional experiences, perceptions, motivation, and classroom participation (Frenzel et al., 2009; Cheng et al., 2022). A recent conceptual review further demonstrates the relevance of emotional contagion to classroom interactions by showing how teachers’ and students’ emotions may be transmitted through facial expressions, vocal characteristics, gestures, and other interpersonal cues (Kong, 2022). Accordingly, the theory provides a basis for understanding how teachers’ recurrent emotional displays may extend beyond individual interactions and become embedded in students’ shared perceptions of the classroom environment.

In addition, control-value theory complements this explanation by addressing how learning environments are associated with students’ achievement emotions and behavioral responses. The theory proposes that students’ emotions and approach or avoidance tendencies depend primarily on their perceptions of control over academic activities and the value assigned to those activities (Pekrun, 2006). Its recent extension explicitly recognizes classroom instruction and social climate as important contextual antecedents of students’ control-value appraisals and achievement emotions (Pekrun, 2024). Consistent with this reasoning, supportive classroom climates have been associated with students’ participation, behavioral adaptation, and willingness to undertake intellectually challenging activities (Gezer, 2023; Olivier et al., 2024). Thus, control-value theory explains why a climate characterized by psychological safety and support may reduce the anticipated emotional cost of failure and be positively associated with academic risk-taking.

Besides, social cognitive theory proposes that behavior emerges through reciprocal relations among environmental conditions, personal capabilities, and behavioral processes (Bandura, 1986). Contemporary applications of the theory emphasize that personal resources influence how students interpret environmental opportunities, regulate their behavior, and sustain goal-directed action (Schunk and DiBenedetto, 2020). Emotional intelligence represents such a personal resource because it assists students in perceiving emotional cues, regulating anxiety, and responding adaptively to challenging situations (Nguyen et al., 2023; Zhou et al., 2024). Therefore, students may respond differently to the same supportive classroom climate depending on their emotional intelligence.

Taken together, these theories explain the complete conditional process proposed in this study: emotional contagion theory explains how teacher emotional display may become embedded in classroom climate; control-value theory explains why classroom climate may be associated with academic risk-taking; and social cognitive theory explains why the strength of this cross-level relationship may vary according to students’ emotional intelligence.

### 2.2 Teacher emotional display and academic risk-taking behavior

Emotions constitute a central yet often underappreciated dimension of instructional practice and are closely associated with how students interpret classroom experiences and engage with learning tasks. Teachers’ emotional displays—manifested through expressions of enthusiasm, encouragement, or warmth (Cheng et al., 2022)—provide salient social signals associated with students’ perceptions of task value and psychological safety. From an emotional contagion perspective (Herrando and Constantinides, 2021), students may internalize the affective cues exhibited by teachers, which may subsequently be associated with their motivation and behavioral engagement. Empirical research has consistently shown that positive teacher emotions are associated with greater student involvement, persistence, and willingness to participate in classroom activities (Cui et al., 2022; Poulou and Denham, 2023). Specifically, teacher enthusiasm has been linked to increased student interest and active participation in higher education settings (Cui et al., 2022; Yan et al., 2023). These findings suggest that emotional expressions are not merely reflective of teachers’ internal states but may also be meaningfully associated with students’ learning-related behaviors.

Moreover, prior studies indicate that emotions experienced in achievement settings are associated with students’ approach or avoidance tendencies. When teachers display positive emotions, they may signal that the environment is safe for exploration, potentially reducing students’ fear of negative evaluation and increasing their readiness to engage with challenging material (Ma et al., 2023). This is particularly relevant for academic risk-taking, which is defined as students’ willingness to attempt challenging academic tasks despite the possibility of error or failure (Clifford, 1991). Conversely, negative or suppressed emotional expressions have been associated with heightened anxiety and greater reluctance to take initiative (Savina and Fulton, 2024). In this sense, teacher emotional display functions as a contextual correlate of students’ willingness to explore uncertain academic territory.

Extending this line of reasoning, research on classroom engagement further suggests that emotionally supportive teaching practices—characterized by positive emotional expression—are associated with active participation and exploratory behaviors (Pi et al., 2022). Such behaviors share conceptual similarities with academic risk-taking, as both involve a willingness to step beyond comfort zones. While existing studies have predominantly examined broad outcomes such as engagement or academic achievement (Alrashidi and Alshammari, 2025; Li et al., 2024), academic risk-taking remains a distinct behavioral construct that may depend on students perceiving that questioning, experimentation, and mistakes are accepted within the learning environment. Recent evidence indicates that supportive teacher–student relationships are associated with students’ innovative behavior (Su et al., 2024), while a positive classroom climate has been linked directly to intellectual risk-taking (Gezer, 2023). These findings provide convergent support for expecting positive teacher emotional displays to be associated with students’ willingness to engage in challenging and uncertain academic activities. Hence, it is hypothesized that:

*H1*: Teacher emotional display is positively associated with students’ academic risk-taking behavior.

### 2.3 Teacher emotional display and classroom climate

Classroom climate reflects students’ shared perceptions of support, trust, cooperation, involvement, and psychological safety within the learning environment (Doll and Dooley, 2024). Although classroom climate emerges collectively, teachers represent an important source of the recurrent interpersonal signals from which students form these shared perceptions. Teachers’ expressions of enthusiasm, encouragement, warmth, frustration, or disappointment may communicate whether participation is valued, mistakes are tolerated, and students can interact without fear of negative evaluation (Cheng et al., 2022; Ma et al., 2023). Teacher emotional display may therefore be associated not only with individual students’ immediate reactions but also with the broader socio-emotional character attributed to the classroom.

Emotional contagion theory provides the principal explanation for this classroom-level relationship. From an emotional contagion perspective, teachers’ affective expressions are not confined to dyadic interactions but may diffuse across the classroom and contribute to a collective emotional tone that students jointly experience (Herrando and Constantinides, 2021; Kong, 2022). Repeated exposure to similar emotional cues may produce affective convergence among classroom members, allowing an initially teacher-originated emotional signal to become embedded in students’ shared environmental perceptions. Positive emotional displays, such as enthusiasm and encouragement, may signal approachability and support, whereas recurrent negative or inconsistent displays may contribute to perceptions of tension or emotional unpredictability. Empirical evidence has connected teacher emotional display and emotional transmission with students’ emotional experiences and perceptions of classroom interactions (Frenzel et al., 2009; Cheng et al., 2022). Teacher emotional labor and display rules have also been associated with the emotional climate experienced within classrooms (Ma et al., 2023). Accordingly, positive teacher emotional display is expected to correspond with a more supportive and psychologically safe classroom climate.

*H2*: Teacher emotional display is positively associated with classroom climate.

### 2.4 Classroom climate and academic risk-taking behavior

A supportive classroom climate is characterized by trust, cooperation, mutual respect, involvement, and psychological safety. These characteristics are particularly relevant to academic risk-taking because attempting a difficult task, proposing an unfamiliar idea, or answering an uncertain question exposes students to the possibility of error and negative evaluation (Clifford, 1991). When students perceive that mistakes are treated as opportunities for learning rather than evidence of incompetence, undertaking challenging academic activities may become more psychologically acceptable. Conversely, a critical, competitive, or unsupportive climate may make students reluctant to reveal incomplete knowledge or attempt tasks with uncertain outcomes.

Control-value theory provides the theoretical basis for the proposed relationship between classroom climate and academic risk-taking. The theory proposes that students’ achievement emotions and approach or avoidance behaviors depend substantially on their perceptions of control over academic activities and the value attached to those activities (Pekrun, 2006, 2024). A supportive climate may strengthen perceived control by providing clear assistance, constructive feedback, and tolerance for mistakes (Olivier et al., 2024). It may simultaneously enhance task value by communicating that participation, exploration, and intellectual effort are appreciated (Pekrun, 2024). By strengthening control and value appraisals while reducing the anticipated emotional cost of failure, a positive classroom climate may be associated with greater willingness to undertake challenging academic tasks.

Empirical research supports this expectation. Positive classroom climates have been associated with participation, persistence, behavioral adaptation, and engagement in complex learning activities (Olivier et al., 2024; Zurbriggen et al., 2023). More directly, Gezer (2023) identified a positive relationship between students’ perceptions of classroom climate and intellectual risk-taking. This evidence indicates that academic risk-taking depends not only on students’ individual preferences but also on whether the classroom environment provides sufficient emotional and interpersonal safety for experimentation. Therefore, students who perceive a more supportive classroom climate are expected to report greater willingness to engage in academically uncertain activities.

*H3*: Classroom climate is positively associated with students’ academic risk-taking behavior.

### 2.5 Mediating role of classroom climate

The preceding arguments establish two theoretically connected relationships: teacher emotional display is expected to be positively associated with classroom climate, and classroom climate is expected to be positively associated with academic risk-taking behavior. Integrating these relationships suggests that classroom climate may function as a collective-level mechanism connecting teachers’ emotional signals with students’ individual behavioral responses. Rather than being associated with academic risk-taking only through students’ immediate reactions, teachers’ recurrent emotional displays may become embedded in shared perceptions of support, cooperation, and psychological safety (Ma et al., 2023), which are subsequently associated with students’ willingness to attempt challenging academic tasks.

This mediating argument integrates emotional contagion theory with control-value theory. Emotional contagion theory explains the first stage of the process: teachers’ recurrent emotional displays may diffuse across classroom interactions and contribute to a shared emotional environment (Herrando and Constantinides, 2021; Kong, 2022). Control-value theory explains the second stage: a supportive classroom environment may strengthen students’ perceptions of control and task value while reducing anxiety surrounding possible failure (Pekrun, 2006, 2024). Together, the theories specify a sequential process through which a classroom-level emotional signal may be associated with an individual-level learning behavior.

Existing studies have often examined teacher behavior and classroom climate as separate correlates of student outcomes, leaving this intervening process insufficiently specified (Erdem and Kaya, 2024; Wang et al., 2025). Research nevertheless indicates that teacher-related emotional and relational conditions are associated with students’ perceptions of the classroom environment (Ma et al., 2023), while classroom climate is associated with motivation, participation, behavioral adaptation, and perceived learning outcomes (Luo and Derakhshan, 2024; Olivier et al., 2024). The present study extends this literature by testing classroom climate as a shared, classroom-level conduit through which teacher emotional display is indirectly associated with students’ academic risk-taking. This multilevel mediation perspective distinguishes the collective environmental mechanism from the individual behavioral outcome and explains how an association originating at the classroom level may extend to students nested within that classroom. Accordingly, the following mediation hypothesis is proposed:

*H4*: Classroom climate mediates the relationship between teacher emotional display and students’ academic risk-taking behavior.

### 2.6 Moderating role of student emotional intelligence

While a supportive classroom climate provides a conducive environment for engagement, students differ in their capacity to interpret and respond to such contextual cues. Student emotional intelligence, defined as the ability to perceive, understand, regulate, and utilize emotions (Wong and Law, 2002), represents a critical individual-level resource for navigating emotionally demanding learning situations. Students with higher emotional intelligence may be better able to recognize supportive cues, manage anxiety, interpret feedback constructively, and maintain adaptive responses when confronted with possible failure. These capabilities are especially relevant to academic risk-taking because attempting unfamiliar or difficult tasks requires students to manage uncertainty, embarrassment, and fear of negative evaluation.

Social cognitive theory provides the theoretical basis for positioning emotional intelligence as a moderator. The theory proposes that behavior reflects reciprocal relations among environmental conditions, personal capabilities, and behavioral processes (Bandura, 1986; Schunk and DiBenedetto, 2020). Accordingly, the same classroom environment may not correspond with identical behavioral responses among all students because personal capabilities affect how environmental opportunities are recognized and utilized. In the present model, classroom climate represents an environmental condition, emotional intelligence represents a personal capability, and academic risk-taking represents the behavioral response.

Empirical research indicates that students with higher emotional intelligence tend to demonstrate stronger emotional regulation, resilience, engagement, and adaptation when confronting academic challenges (Năstasă et al., 2022; Valverde-Janer et al., 2023). Wang and Wang (2024) has also assessed classroom climate and emotional intelligence with students’ academic emotions and learning experiences, indicating that contextual and emotional resources may operate jointly rather than independently. However, limited research has examined whether emotional intelligence changes the strength of the relationship between a shared classroom climate and students’ academic risk-taking.

Students with higher emotional intelligence may be particularly capable of interpreting trust, support, and tolerance for mistakes as signals that challenging participation is psychologically acceptable. They may also regulate the anxiety accompanying possible failure and use constructive feedback without interpreting it as a threat to their competence (Nguyen et al., 2023). Students with lower emotional intelligence, by contrast, may find it more difficult to recognize or utilize the same contextual support. Therefore, the positive association between classroom climate and academic risk-taking is expected to be stronger among students with higher emotional intelligence. This argument positions emotional intelligence not merely as a direct correlate of academic outcomes but as an individual-level boundary condition of a cross-level environmental relationship.

*H5*: Student emotional intelligence moderates the relationship between classroom climate and academic risk-taking behavior, such that the positive relationship is stronger at higher levels of emotional intelligence.

### 2.7 Conditional indirect effect of student emotional intelligence

The preceding hypotheses propose that teacher emotional display is associated with students’ academic risk-taking through classroom climate and that the relationship between classroom climate and academic risk-taking varies according to student emotional intelligence. Integrating these arguments suggests a conditional indirect process: the indirect association between teacher emotional display and academic risk-taking through classroom climate may be stronger for students who possess greater emotional intelligence.

This conditional process integrates the three theoretical perspectives underlying the study. Emotional contagion theory explains how teachers’ recurrent emotional displays may diffuse across classroom interactions and become embedded in a shared classroom climate (Herrando and Constantinides, 2021; Kong, 2022). Control-value theory explains why that climate may be associated with academic risk-taking by strengthening students’ perceived control and task value while reducing the anticipated emotional cost of failure (Pekrun, 2006, 2024). Social cognitive theory adds that the behavioral relevance of an environmental resource depends partly on individuals’ capabilities to interpret and utilize it (Bandura, 1986; Schunk and DiBenedetto, 2020). Emotional intelligence therefore determines the condition under which the classroom-level pathway may be more strongly associated with students’ individual behavior.

When emotional intelligence is high, students may be more capable of recognizing the support conveyed by a positive classroom climate, regulating anxiety associated with mistakes, and converting contextual reassurance into willingness to attempt challenging tasks. Consequently, the indirect association between teacher emotional display and academic risk-taking through classroom climate should be stronger. When emotional intelligence is low, students may be less able to recognize or utilize the same classroom-level support, resulting in a weaker indirect association.

This argument extends research that has generally examined emotional intelligence as a direct correlate of academic performance, resilience, or engagement (Năstasă et al., 2022; Nguyen et al., 2023; Zhou et al., 2024). It also moves beyond studies treating classroom climate as having a uniform relationship with all students. By conceptualizing emotional intelligence as a Level 1 moderator of the Level 2 classroom climate–Level 1 academic risk-taking relationship, the present study identifies when and for whom the classroom-level emotional process is most strongly associated with adaptive academic behavior. Accordingly, the following hypothesis is proposed:

*H6*: Student emotional intelligence moderates the indirect relationship between teacher emotional display and academic risk-taking behavior through classroom climate, such that the positive indirect relationship is stronger at higher levels of emotional intelligence.

## 3 Methodology

### 3.1 Research design, sampling, and participants

Data were collected from undergraduate students enrolled in business management, information technology, and engineering programs across universities in China. A multistage sampling approach was used to capture variation across academic disciplines and classrooms while maintaining comparability in instructional settings.

A total of 530 paper-based questionnaires were distributed across 75 classrooms, of which 463 were returned, yielding a response rate of 87.4%. After excluding 44 questionnaires with substantial missing data or invariant response patterns, 419 usable questionnaires were retained. The final usable response rate was therefore 79.1% of the initially distributed questionnaires and 90.5% of those returned. Each questionnaire included a cover letter explaining the study’s purpose, voluntary nature, confidentiality protections, and participants’ right to withdraw.

A three-wave time-lagged design was employed, with approximately 3 weeks between consecutive waves. At Time 1, participants assessed teacher emotional display and provided demographic information. At Time 2, classroom climate and student emotional intelligence were measured. At Time 3, participants reported their academic risk-taking behavior. Questionnaires were matched across the three waves using anonymous identification codes. Temporal separation, anonymity, and separation of predictor and outcome measurements were employed to reduce consistency motifs, evaluation apprehension, and social-desirability bias.

The final sample comprised 419 students nested within 68 classrooms, with an average cluster size of 6.16 students. Of the participants, 218 (52.0%) were male and 201 (48.0%) were female. Regarding age, 62% were between 18 and 20 years, 31% were between 21 and 22 years, and 7% were older than 22 years. The sample included first-year (28%), second-year (34%), third-year (26%), and fourth-year students (12%).

### 3.2 Measures, adaptation, and translation

All constructs were measured using established multi-item scales. Following Brislin’s (1980) procedure, the original English items were translated into Mandarin and independently back-translated into English. The original and back-translated versions were compared for semantic and conceptual equivalence, and discrepancies were resolved before questionnaire administration. Item wording was adapted minimally to refer specifically to university classroom experiences. All items were rated on a five-point Likert scale ranging from 1 (*strongly disagree*) to 5 (*strongly agree*).

Teacher emotional display was measured using nine items adapted from Frenzel et al. (2009). The items captured teachers’ displayed enthusiasm, supportiveness, and negative emotional expressions during instruction. Representative items included “My teacher shows enthusiasm while teaching” and “My teacher expresses frustration during class.” Negatively worded items were reverse-coded so that higher scores represented more positive teacher emotional display.

Classroom climate was assessed using eight items adapted from the Classroom Environment Scale (Moos and Trickett, 1974) and subsequent educational applications (Fraser, 2012). The scale assessed students’ shared perceptions of support, involvement, cooperation, and psychological safety. A representative item was “Students in this class support and help each other.”

Student emotional intelligence was measured using the 16-item Wong and Law Emotional Intelligence Scale (Wong and Law, 2002). The scale assessed self-emotion appraisal, others’ emotion appraisal, emotion regulation, and emotion utilization. Representative items included “I have a good understanding of my own emotions” and “I am able to control my temper when I face difficulties.”

Academic risk-taking behavior was measured using six items adapted from Clifford (1991). The items assessed students’ willingness to attempt challenging academic tasks despite the possibility of error or failure. Representative items included “I attempt difficult questions even if I might make mistakes” and “I prefer challenging tasks even when they are risky.”

### 3.3 Analytical strategy

The analyses were conducted in Mplus 8.10 using multilevel structural equation modeling with robust maximum likelihood estimation. Before model estimation, the data were screened for missing values, response-pattern irregularities, influential observations, normality, linearity, homoscedasticity, and multicollinearity. Absolute skewness values ranged from 0.21 to 0.68, while absolute kurtosis values ranged from 0.18 to 0.76, indicating no serious distributional departures. Inspection of residual plots supported linearity and homoscedasticity. Variance inflation factors ranged from 1.18 to 1.62, and tolerance values ranged from 0.62 to 0.85, indicating that multicollinearity was not a concern.

The psychometric properties of the measures were examined before testing the structural model. Internal consistency was assessed using Cronbach’s alpha and composite reliability, with values of 0.70 or higher indicating acceptable reliability. Convergent validity was supported when standardized factor loadings were statistically significant and exceeded 0.60, composite reliability exceeded 0.70, and average variance extracted exceeded 0.50. Discriminant validity was evaluated using the Fornell–Larcker criterion and by confirming that average variance extracted exceeded maximum shared variance and average shared variance.

Model fit was evaluated using chi-square, the chi-square-to-degrees-of-freedom ratio, the comparative fit index, the Tucker–Lewis index, the root mean square error of approximation, and the standardized root mean square residual at the within- and between-classroom levels. Values of *χ*^2^/df below 3, CFI and TLI values of at least 0.90, and RMSEA and SRMR values below 0.08 were considered indicative of acceptable model fit.

Because teacher emotional display and classroom climate represented shared classroom-level constructs, their aggregation was evaluated using ICC(1), ICC(2), within-group agreement, and design effects (Table 1). Teacher emotional display produced ICC(1) = 0.29, ICC(2) = 0.72, median (*r*_wg_) = 0.84, and a design effect of 2.50. Classroom climate produced ICC(1) = 0.32, ICC(2) = 0.74, median (*r*_wg_) = 0.87, and a design effect of 2.65. These statistics indicated meaningful between-classroom variation, acceptable reliability of classroom means, and strong within-classroom agreement, supporting aggregation to Level 2. Student emotional intelligence and academic risk-taking behavior were retained at Level 1.

**Table 1:** Aggregation statistics.

Construct	ICC(1)	ICC(2)	Median (r_wg_)	Design effect	Decision
Teacher emotional display	0.29	0.72	0.84	2.50	Aggregated
Classroom climate	0.32	0.74	0.87	2.65	Aggregated

Average classroom size = 6.16. Design effect was calculated as 1 + (average classroom size – 1) x ICC(1).

Teacher emotional display and classroom climate were grand-mean centered, whereas student emotional intelligence was group-mean centered. Gender, age, and grade level were entered as Level 1 control variables. H1–H3 were examined through their respective multilevel structural paths. H4 was evaluated as a 2–2–1 indirect effect of teacher emotional display on academic risk-taking through classroom climate. H5 was tested through the classroom climate × student emotional intelligence cross-level interaction. Significant interactions were interpreted using conditional slopes at one standard deviation below the mean, the mean, and one standard deviation above the mean of emotional intelligence. H6 was evaluated by estimating conditional indirect effects at the same moderator values. Bias-corrected 95% confidence intervals were obtained using 5,000 cluster-level bootstrap samples, with classrooms resampled to preserve the nested data structure.

Common-method variance was assessed using procedural and statistical remedies. Harman’s single-factor test indicated that the first unrotated factor accounted for 28.4% of the total variance. In addition, a common-latent-factor model was compared with the hypothesized measurement model. The inclusion of the common latent factor produced only a small change in model fit (ΔCFI = 0.005), while changes in standardized substantive loadings ranged from 0.012 to 0.074. These results indicated that common-method variance was unlikely to account for the observed relationships.

## 4 Results

Preliminary analyses indicated that the statistical assumptions were adequately satisfied. Absolute skewness values ranged from 0.21 to 0.68, and absolute kurtosis values ranged from 0.18 to 0.76, indicating no substantial departure from normality. Inspection of scatterplots and residual plots supported linearity and homoscedasticity. Variance inflation factors ranged from 1.18 to 1.62, with tolerance values ranging from 0.62 to 0.85, indicating that multicollinearity was not a concern. The aggregation statistics also supported the multilevel specification. Teacher emotional display produced ICC(1) = 0.29, ICC(2) = 0.72, median rwg = 0.84, and a design effect of 2.50, whereas classroom climate produced ICC(1) = 0.32, ICC(2) = 0.74, median rwg = 0.87, and a design effect of 2.65.

Descriptive statistics and correlations among the study variables are presented in Table 2. The results indicate that teacher emotional display, classroom climate, student emotional intelligence, and academic risk-taking behavior are all positively associated with one another. In particular, teacher emotional display shows a strong positive correlation with classroom climate (*r* = 0.521, *p* < 0.01) and a moderate relationship with academic risk-taking behavior (*r* = 0.401, *p* < 0.01), suggesting that emotionally expressive teaching is linked to both a supportive classroom environment and students’ willingness to engage in challenging tasks. Classroom climate also demonstrates a significant positive association with academic risk-taking behavior (*r* = 0.472, *p* < 0.01), providing preliminary support for its mediating role. Student emotional intelligence is positively related to both classroom climate (*r* = 0.356, *p* < 0.01) and academic risk-taking behavior (*r* = 0.389, *p* < 0.01), indicating its relevance as a boundary condition in the model.

**Table 2:** Descriptive statistics and correlations.

Variables	M	SD	1	2	3	4	5	6	7
1. Gender (1 = male)	0.520	0.500	–						
2. Age	20.340	1.210	0.042	–					
3. Grade level	2.310	0.880	0.065	0.412**	–				
4. Teacher emotional display (TED)	3.780	0.640	0.081	0.053	0.072	–			
5. Classroom climate (CC)	3.910	0.580	0.067	0.061	0.089	0.521**	–		
6. Student emotional intelligence (SEI)	3.650	0.620	0.094	0.102*	0.075	0.318**	0.356**	–	
7. Academic risk-taking behavior (ARTB)	3.720	0.600	0.058	0.087	0.066	0.401**	0.472**	0.389**	–

*N* = 419 students nested within 68 classrooms; *p < 0.05, **p < 0.01.

In contrast, demographic variables such as gender, age, and grade level show relatively weak and mostly non-significant correlations with the main constructs. Gender, age, and grade level were included as Level 1 control variables in the structural model. Their associations with academic risk-taking were not statistically significant: gender, *B* = 0.024, SE = 0.032, *p* = 0.453; age, *B* = 0.018, SE = 0.021, *p* = 0.391; and grade level, *B* = 0.029, SE = 0.024, *p* = 0.227. The inclusion of these control variables did not materially change the magnitude or statistical significance of the hypothesized relationships.

Table 3 reports the results of the measurement model assessment, confirming satisfactory convergent and discriminant validity. All constructs exhibit strong internal consistency, with Cronbach’s alpha values ranging from 0.881 to 0.910 and composite reliability values ranging from 0.884 to 0.913, exceeding the recommended threshold of 0.70. Convergent validity is established as average variance extracted values range from 0.603 to 0.677, all above the acceptable cutoff of 0.50. Discriminant validity is supported through multiple criteria. First, the square root of the average variance extracted for each construct (diagonal elements) exceeds the corresponding inter-construct correlations, satisfying the Fornell–Larcker criterion. Second, for all constructs, average variance extracted values are greater than both maximum shared variance and average shared variance, further indicating that each construct captures a distinct concept. Collectively, these results demonstrate that the measurement model possesses adequate reliability and validity, providing a robust foundation for subsequent structural model analysis.

**Table 3:** Reliability, convergent validity, and discriminant validity.

Variables	Alpha	CR	AVE	MSV	ASV	TED	CC	SEI	ARTB
TED	0.881	0.884	0.603	0.272	0.178	0.777			
CC	0.897	0.901	0.645	0.272	0.207	0.521	0.803		
SEI	0.910	0.913	0.677	0.151	0.126	0.318	0.356	0.823	
ARTB	0.884	0.887	0.611	0.223	0.178	0.401	0.472	0.389	0.782

To further establish discriminant validity, a series of confirmatory factor analyses were conducted comparing the hypothesized four-factor model with alternative models. As shown in Table 4, the four-factor model exhibited a good fit to the data (*χ*^2^ = 512.384, df = 224, *χ*^2^/df = 2.288, CFI = 0.952, TLI = 0.944, RMSEA = 0.056). In contrast, alternative models combining constructs demonstrated substantially poorer fit, with notable declines in CFI and TLI and increases in RMSEA. These results indicate that the proposed four-factor structure provides the best representation of the data, thereby supporting the discriminant validity of the constructs.

**Table 4:** Confirmatory factor analysis model comparison.

Model	*χ* ^2^	df	*χ*^2^/df	CFI	TLI	RMSEA
Four-factor model (TED, CC, SEI, ARTB)	512.384	224	2.288	0.952	0.944	0.056
Three-factor model (TED + CC combined)	873.615	227	3.847	0.892	0.874	0.084
Two-factor model (TED + CC + SEI combined)	1264.902	229	5.525	0.821	0.793	0.112
One-factor model (all variables combined)	1987.431	230	8.641	0.702	0.661	0.158

The hypothesized structural model demonstrated acceptable fit to the data: *χ*^2^ = 538.216, df = 238, *χ*^2^/df = 2.261, CFI = 0.949, TLI = 0.941, RMSEA = 0.055, SRMR-within = 0.043, and SRMR-between = 0.061. As reported in Table 5, teacher emotional display was positively associated with academic risk-taking behavior, *B* = 0.183, SE = 0.052, 95% CI [0.082, 0.284], supporting H1. Teacher emotional display was also positively associated with classroom climate, *B* = 0.521, SE = 0.041, 95% CI [0.441, 0.601], supporting H2. Classroom climate was positively associated with academic risk-taking behavior, *B* = 0.347, SE = 0.049, 95% CI [0.251, 0.443], supporting H3.

**Table 5:** Structural paths.

Hypothesis	Path	*B*	SE	95% CI (LL, UL)	Decision
Direct effects					
H1	TED → ARTB	0.183	0.052	[0.082, 0.284]	Supported
H2	TED → CC	0.521	0.041	[0.441, 0.601]	Supported
H3	CC → ARTB	0.347	0.049	[0.251, 0.443]	Supported
Indirect effect (mediation)					
H4	TED → CC → ARTB	0.181	0.036	[0.113, 0.253]	Supported
Interaction effect (moderation)					
H5	CC × SEI → ARTB	0.129	0.031	[0.068, 0.190]	Supported
Conditional indirect effects (moderated mediation)					
H6	Low SEI (−1 SD)	0.112	0.030	[0.061, 0.178]	Supported
	Mean SEI	0.181	0.036	[0.113, 0.253]	Supported
	High SEI (+1 SD)	0.254	0.046	[0.162, 0.341]	Supported
Index of moderated mediation		0.067	0.021	[0.029, 0.114]	Supported

The indirect association between teacher emotional display and academic risk-taking through classroom climate was statistically significant, *B* = 0.181, SE = 0.036, 95% CI [0.113, 0.253], supporting H4. The interaction between classroom climate and student emotional intelligence was also statistically significant, *B* = 0.129, SE = 0.031, 95% CI [0.068, 0.190], supporting H5 (Figure 2).

**Figure 2:**
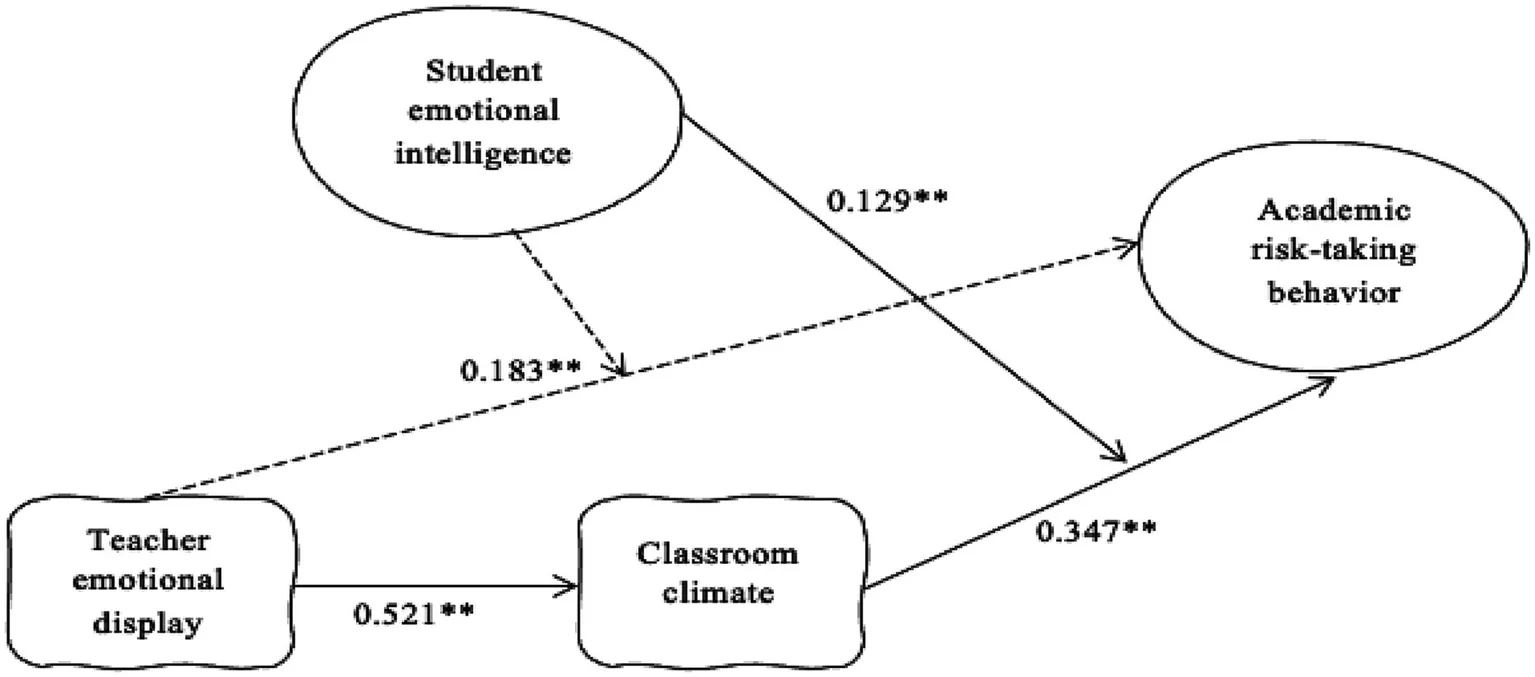
Model results. **p < 0.01.

Simple-slope analyses showed that classroom climate was positively associated with academic risk-taking at low, mean, and high levels of student emotional intelligence. At one standard deviation below the mean of emotional intelligence, the relationship was positive and statistically significant, *B* = 0.218, SE = 0.066, 95% CI [0.089, 0.347], *p* = 0.001. At the mean level of emotional intelligence, the relationship remained positive and statistically significant, *B* = 0.347, SE = 0.049, 95% CI [0.251, 0.443], *p* < 0.001. At one standard deviation above the mean of emotional intelligence, the relationship was stronger, *B* = 0.476, SE = 0.061, 95% CI [0.356, 0.596], *p* < 0.001. As illustrated in Figure 3, the positive relationship between classroom climate and academic risk-taking was stronger among students with higher emotional intelligence.

**Figure 3:**
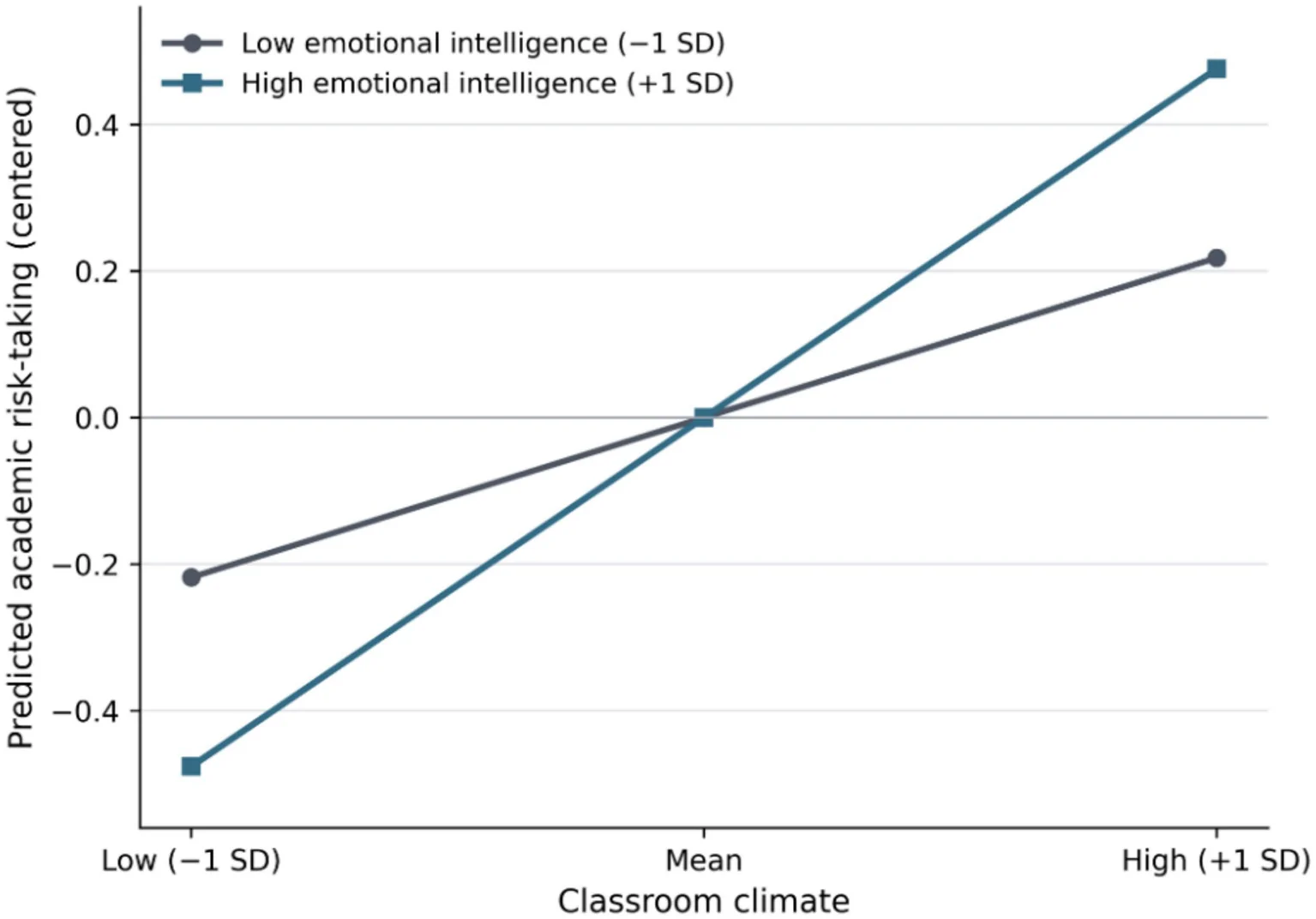
Moderating effect of student emotional intelligence on the relationship between classroom climate and academic risk-taking behavior.

The conditional indirect association between teacher emotional display and academic risk-taking through classroom climate was significant at all three levels of emotional intelligence. At one standard deviation below the mean, the conditional indirect association was *B* = 0.112, SE = 0.030, 95% CI [0.061, 0.178]. At the mean level, the conditional indirect association was B = 0.181, SE = 0.036, 95% CI [0.113, 0.253]. At one standard deviation above the mean, the conditional indirect association increased to *B* = 0.254, SE = 0.046, 95% CI [0.162, 0.341]. The index of moderated mediation was statistically significant, *B* = 0.067, SE = 0.021, 95% CI [0.029, 0.114], confirming that the indirect association became stronger as emotional intelligence increased. Thus, H6 was supported.

The multilevel structural model explained 27.1% of the between-classroom variance in classroom climate, 38.4% of the within-classroom variance in academic risk-taking, and 46.5% of the between-classroom variance in academic risk-taking. As shown in Table 6, the random-intercept variance for academic risk-taking was statistically significant, τ00 = 0.046, SE = 0.015, *p* = 0.002, indicating that average levels of academic risk-taking differed across classrooms even after accounting for the predictors. The random-slope variance for student emotional intelligence was also statistically significant, τ11 = 0.012, SE = 0.006, *p* = 0.046, indicating that the relationship between emotional intelligence and academic risk-taking varied across classrooms. However, the intercept–slope covariance was not statistically significant, τ01 = 0.006, SE = 0.004, *p* = 0.134, suggesting that classrooms with higher average levels of academic risk-taking did not systematically exhibit stronger or weaker emotional intelligence slopes.

**Table 6:** Multilevel structural model diagnostics.

Outcome or parameter	Estimate	SE	*p*
Classroom climate: between-level *R*^2^	0.271	–	–
Academic risk-taking: within-level *R*^2^	0.384	–	–
Academic risk-taking: between-level *R*^2^	0.465	–	–
ARTB random-intercept variance, *τ_00_*	0.046	0.015	0.002
SEI random-slope variance, *τ_11_*	0.012	0.006	0.046
Intercept–slope covariance, *τ_01_*	0.006	0.004	0.134

As reported in Table 7, the hypothesized moderated model demonstrated better fit than the model excluding the interaction. The reduction in chi-square was statistically significant, Δ*χ*^2^(1) = 16.840, *p* < 0.001. The hypothesized model also demonstrated higher CFI and TLI values, lower RMSEA, SRMR-W, and SRMR-B values, and lower AIC and BIC values. Collectively, these results indicate that including the interaction between classroom climate and student emotional intelligence significantly improved the model and provided additional support for the proposed cross-level moderation effect.

**Table 7:** Structural model comparison.

Model	*χ* ^2^	df	CFI	TLI	RMSEA	SRMR-W	SRMR-B	AIC	BIC
Model excluding interaction	555.056	239	0.946	0.938	0.056	0.049	0.064	28,321.460	28,704.780
Hypothesized moderated model	538.216	238	0.949	0.941	0.055	0.043	0.061	28,306.620	28,693.980

## 5 Discussion and implications

The findings of this study provide a coherent and theory-consistent account of how teacher emotional display is associated with students’ academic risk-taking behavior through both direct and indirect pathways. The positive association between teacher emotional display and academic risk-taking suggests that emotionally expressive teaching functions as a salient contextual cue associated with reduced uncertainty and greater willingness among students to engage in challenging academic tasks. This aligns with prior research showing that teachers’ positive emotional expressions, such as enthusiasm and support, are associated with student engagement and willingness to participate in learning activities (Cui, 2022; Cui et al., 2022; Yan et al., 2023). While earlier studies have primarily focused on outcomes such as engagement and achievement, the present findings extend this line of inquiry by identifying academic risk-taking as a distinct behavioral outcome associated with teacher emotional display, thereby broadening the scope of emotional associations in classroom settings.

Regarding H2, teacher emotional display was positively associated with classroom climate. This finding supports emotional contagion theory by indicating that teachers’ recurrent expressions of enthusiasm, encouragement, and support may extend beyond immediate teacher–student interactions and become reflected in students’ shared perceptions of the classroom environment. The result is consistent with research connecting teachers’ emotional expressions and emotionally supportive behaviors with students’ perceptions of classroom interactions and collective emotional conditions (Cheng et al., 2022; Frenzel et al., 2009; Ma et al., 2023). By locating this relationship at the classroom level, the study further indicates that teacher emotional display may be associated not only with individual student reactions but also with the broader climate jointly experienced by classroom members. Future research could strengthen the assessment of this classroom-level emotional process by using multimodal sentiment-analysis approaches to capture teachers’ verbal, facial, and vocal emotional signals more comprehensively (Cai et al., 2026).

Regarding H3, classroom climate was positively associated with students’ academic risk-taking behavior. This finding is consistent with control-value theory, which suggests that supportive classroom conditions may strengthen students’ perceived control and task value while reducing the anticipated emotional cost of mistakes or failure (Pekrun, 2006, 2024). It also accords with evidence linking positive classroom climates with participation, behavioral adaptation, and intellectual risk-taking (Gezer, 2023; Olivier et al., 2024; Zurbriggen et al., 2023). The result therefore suggests that students may be more willing to attempt difficult tasks, propose unfamiliar ideas, and answer uncertain questions when the classroom is perceived as supportive, cooperative, and tolerant of mistakes.

Moreover, the mediating role of classroom climate highlights the importance of shared environmental perceptions in linking teacher behaviors with student outcomes. The results indicate that teacher emotional display is positively associated with a supportive and collaborative classroom climate, which is subsequently associated with greater academic risk-taking. This is consistent with previous studies suggesting that classroom climate serves as a critical mechanism linking instructional practices to student motivation and participation (Doll and Dooley, 2024; Erdem and Kaya, 2024; Gezer, 2023; Peiris et al., 2022). However, the current study advances prior knowledge by demonstrating that this mechanism is evident not only at the individual level but also as a collective classroom phenomenon, reinforcing the multilevel nature of educational processes. In doing so, it provides empirical support for the argument that emotional signals from teachers may become reflected in the broader classroom environment and be associated with students’ behavioral tendencies through shared experiences rather than isolated perceptions. Future studies could employ graph- and network-based analytical approaches to examine how emotional cues and supportive interactions circulate among classroom members and become reflected in a collective classroom climate (Lyu et al., 2026).

The moderating role of student emotional intelligence further refines this understanding by revealing that the strength of the relationship between classroom climate and academic risk-taking varies according to students’ ability to interpret and regulate emotional information. The findings show that the positive relationship between classroom climate and academic risk-taking is stronger for students with higher emotional intelligence, which is consistent with prior research highlighting emotional intelligence as a key personal resource associated with adaptive learning behaviors (Al-Mamary and Alshallaqi, 2022; Shah et al., 2025). While existing studies have established the direct benefits of emotional intelligence for academic performance and well-being, the present study extends this literature by positioning emotional intelligence as a boundary condition under which the strength of students’ responses varies to environmental cues. This underlines the importance of considering individual differences in emotional processing when examining relationships involving classroom dynamics.

Finally, the moderated mediation results integrate these insights by indicating that the indirect association between teacher emotional display and academic risk-taking through classroom climate is contingent upon students’ emotional intelligence. This finding advances theoretical understanding by indicating that emotional and environmental processes are not only interconnected but also conditional in nature. In contrast to prior studies that have examined mediation or moderation in isolation (Fang et al., 2025; Li et al., 2025; Wang et al., 2022; Wang and Liu, 2026), the present research offers a more comprehensive framework that captures the interplay between teacher behavior, classroom context, and student capability. By doing so, it contributes to the literature by extending emotional contagion, control-value, and social cognitive perspectives into a unified multilevel model, providing a richer explanation of the conditions under which academic risk-taking is more strongly associated with classroom emotional processes.

Subsequently, the study presents noteworthy practical implications for educators, institutions, and policymakers seeking to foster adaptive learning behaviors among students. The findings suggest that teacher emotional display is not merely a personal or stylistic attribute but may represent a strategic pedagogical resource associated with supportive classroom dynamics and student engagement. Teachers should be encouraged to consciously express positive emotions such as enthusiasm, encouragement, and support, as these signals may be associated with a psychologically safe and motivating classroom climate. Training programs and professional development initiatives may incorporate emotional awareness and regulation skills, supporting teachers in managing and utilizing their emotional expressions more effectively in instructional settings. Such programs may also incorporate teacher caring and self-efficacy because these relational and personal resources may complement emotional expressiveness in supporting positive classroom experiences (Tang et al., 2026).

In addition, the mediating role of classroom climate highlights the importance of cultivating an environment characterized by trust, openness, and collaboration. Educational institutions should prioritize practices that promote inclusive and supportive classroom interactions, such as cooperative learning activities and constructive feedback mechanisms, which may reduce students’ fear of failure and support academic risk-taking. At the same time, the moderating influence of student emotional intelligence indicates that not all students benefit equally from a positive classroom climate. Therefore, integrating emotional intelligence development into curricula through targeted interventions, workshops, or reflective exercises may strengthen students’ ability to interpret and respond to emotional cues, thereby increasing the potential benefits of supportive environments. Collectively, these implications emphasize that effective teaching extends beyond content delivery to include the intentional management of emotional and contextual factors associated with student behavior.

## 6 Limitations and directions for future research

Despite offering meaningful insights, this study is subject to several limitations that open avenues for future research. First, although a time-lagged design was employed to mitigate common method bias, the data were primarily collected through student self-reports, which may still introduce perceptual biases. Future studies could adopt multi-source data, such as teacher-reported emotional display or peer evaluations of classroom climate, to enhance the robustness of findings. Additionally, incorporating objective or behavioral measures of academic risk-taking, such as participation records or task-selection patterns, would further strengthen validity.

Second, the study was conducted within the context of Chinese higher education, which may limit the generalizability of the findings to other cultural or educational settings. Cultural norms are associated with differences in emotional expression, classroom interactions, and students’ responses to risk, suggesting that the observed relationships may vary across contexts. Future research could employ cross-cultural or comparative designs to examine whether these dynamics hold in different institutional and cultural environments, thereby extending the external validity of the model.

Third, while the multilevel design accounts for variation at the classroom level, the study focuses on a specific set of variables and does not account for other potentially relevant contextual or individual factors. For instance, teacher characteristics such as experience, teaching style, or leadership approach, as well as student traits like motivation, personality, or fear of failure, may also be associated with academic risk-taking behavior. Future research could integrate these variables into more complex models to provide a more comprehensive understanding of the phenomenon.

Finally, the present study adopts a linear modeling approach to examine relationships among variables, which may overlook potential configurational or non-linear relationships. Future studies could employ alternative analytical techniques, such as fuzzy-set qualitative comparative analysis, to explore multiple configurations associated with academic risk-taking. Experimental designs could also be employed to permit stronger causal inferences. Longitudinal research designs extending beyond three time points would also be valuable in examining changes in emotional processes and behavioral outcomes over time.

## 7 Conclusion

In conclusion, this study offers a comprehensive multilevel understanding of the relationships among teacher emotional display, classroom climate, student emotional intelligence, and students’ academic risk-taking behavior by integrating contextual and individual mechanisms. The findings indicate that teacher emotional display is directly associated with students’ willingness to engage in challenging academic tasks and indirectly associated with academic risk-taking through a supportive classroom climate. Moreover, the strength of this indirect relationship varies according to students’ emotional intelligence, highlighting the importance of individual differences in interpreting and responding to classroom dynamics. By combining direct, mediating, and moderating pathways within a unified framework, the study advances theoretical and empirical insights into emotional processes in education. Overall, the results suggest that academic risk-taking is associated not only with instructional practices but also with the emotional and environmental conditions present within the classroom.

## Data Availability

The raw data supporting the conclusions of this article will be made available by the authors, without undue reservation.
